# Race Differences and Depressive Symptoms Among Community-Dwelling Older Americans

**DOI:** 10.1093/geroni/igaa057.323

**Published:** 2020-12-16

**Authors:** Ethan Siu Leung Cheung, Ada Mui

**Affiliations:** Columbia University, New York, New York, United States

## Abstract

Using data from the Wave 3 National Social Life, Health and Aging Project, this study examines cognition, stress and social support factors associated with depressive symptomatology among older White (n=2356) and Black/African Americans (n=473) living in the community. Bivariate analyses suggest that Whites were slightly older than Blacks [(M(SD) = 73 .24(8.18) and 72.52(8.69); 71)]; and had higher unmarried status (66.58% vs. 43.76%). In terms of cognitive functioning, Whites scored significantly higher than Blacks [Mean (SD) of MoCA Short Form were 10.44(3.06) and 7.75.0(3.33)]. There was race difference in depressive symptoms experienced (CESD Short Form: M(SD) = 20.99(4.01) for Whites; M(SD) = 21.35(4.33) for Blacks). In order to identify predictors of depression, multiple hierarchical regressions were performed. Results showed that race had significant independent effect and multiplicative effect with IADL impairment in explaining depression scores. To identify predicators for each racial group, parallel regression analyses were conducted and two models were significant. Findings show that unmarried status and IADL impairment were common predictors of depressive symptoms for the two groups, and the impact of both variables were stronger for Blacks (for unmarried status; b =-1.42 vs. -.52; for IADL impairment b = .23 vs. .13). For Whites, other unique predictors of depression were male gender, lower income, more ADL impairment, higher stress, less socialization and poor friendship quality. For Blacks, the only unique predictor of depressive symptoms was being younger age. The different correlates of depression for White and Black elders provide new insight into the design of race-sensitive interventions.

